# Genome-wide case-control study in GAW17 using coalesced rare variants

**DOI:** 10.1186/1753-6561-5-S9-S110

**Published:** 2011-11-29

**Authors:** Libo Wang, Vitara Pungpapong, Yanzhu Lin, Min Zhang, Dabao Zhang

**Affiliations:** 1 Department of Statistics, Purdue University, West Lafayette, IN 47907, USA

## Abstract

Genome-wide association studies have successfully identified numerous loci at which common variants influence disease risks or quantitative traits of interest. Despite these successes, the variants identified by these studies have generally explained only a small fraction of the variations in the phenotype. One explanation may be that many rare variants that are not included in the common genotyping platforms may contribute substantially to the genetic variations of the diseases. Next-generation sequencing, which would better allow for the analysis of rare variants, is now becoming available and affordable; however, the presence of a large number of rare variants challenges the statistical endeavor to stably identify these disease-causing genetic variants. We conduct a genome-wide association study of Genetic Analysis Workshop 17 case-control data produced by the next-generation sequencing technique and propose that collapsing rare variants within each genetic region through a supervised dimension reduction algorithm leads to several macrovariants constructed for rare variants within each genetic region. A simultaneous association of the phenotype to all common variants and macrovariants is undertaken using a linear discriminant analysis using the penalized orthogonal-components regression algorithm. The results suggest that the proposed analysis strategy shows promise but needs further development.

## Background

Although genome-wide association studies (GWAS) are becoming a powerful tool to discover causal genes of common diseases, it has been hotly debated whether common diseases are caused by common variants or multiple rare variants. Nowadays, more evidence supporting the role of rare variants in disease association (especially for Mendelian disorders) can be found in the literature. Through the advances in next-generation sequencing techniques, it is possible to incorporate rare variants into association studies. In GWAS, the ability to detect an association at a particular single-nucleotide polymorphism (SNP) decreases with the minor allele frequency (MAF) of that SNP; as a result, studies have so far been underpowered to detect associations with rare variants, and so alternative approaches are required.

A natural approach is a collapsing strategy in which rare variants within a defined group are collapsed into a single variant. Individually, low-frequency variants are rare, but within aggregates they may be common enough to account for variations in common traits, which is the basic idea behind the collapsing methods [1]. Li and Leal [2] proposed collapsing rare variants within each genetic region by indicating the presence of the minor allele. Madsen and Browning [3] then proposed a weighted-sum statistic in which variants were weighted according to their frequency in the unaffected sample, with low-frequency variants being weighted more heavily. With most of the existing collapsing methods using an unsupervised dimension reduction algorithm, at this point we propose to collapse rare variants within each genetic region through a partial least-squares (PLS) regression, which is a supervised dimension reduction algorithm that leads to several “macrovariants” constructed for the rare variants within each genetic region [4].

A simultaneous study of the phenotypic association of all common variants and macrovariants will generate robust hypotheses for subsequent biological investigations.

With the available common variants and macrovariants, we conduct the genome-wide case-control study by using a linear discriminant analysis (LDA). The LDA is implemented using the penalized orthogonal-components regression (POCRE) algorithm, which sequentially constructs sparsely loaded orthogonal components with proper regularization. The superior performance of the POCRE algorithm in fitting regression models with large *p* and small *n* data, as shown by Zhang et al. [5], allows us to reliably identify the potential disease-causing genetic variants out of a great number of candidates. We applied the POCRE algorithm to the genome-wide association study of the binary trait in the Genetic Analysis Workshop 17 (GAW17) data using both common variants and macrovariants constructed using a PLS regression.

## Methods

### Collapsing rare variants within each genetic region

Here, we use the PLS regression, which is a supervised dimension reduction approach, to help collapse the rare variants within each genetic region. Consider the following model for each gene:(1)

where *Y* is the phenotype vector and {*X*_1_, …, *X_k_*} are the genotypes of *k* rare variants within the gene. The PLS regression is used to find a set of components that are linear combinations of the *X_j_* with the constraint that these components explain as much as possible the covariance between *Y* and *X*. Cross validation is used to determine the optimal number of components for each gene.

### Linear discriminant analysis using penalized orthogonal-components regression

As shown by Zhang et al. [5], the POCRE algorithm sequentially constructs orthogonal components to maximize, upon standardization, their correlation to the response residuals; at the same time, the algorithm uses a penalization by means of empirical Bayes thresholding [6] to effectively identify sparse predictors for each component. The POCRE algorithm is computationally efficient because of its sequential construction of leading sparse principal components. In addition, this construction offers other distinct properties, such as the ability to group highly correlated predictors and to allow for collinear or nearly collinear predictors.

Consider the following multiple linear regression:(2)

where *Y* is the phenotype vector of length *n*, *X* is an *n* × *p* genotype matrix, *β_j_* is the additive effect of that genetic variant, *j* = 1, …, *p*, *n* is the number of individuals, and *p* is the number of SNPs. We then further assume that *Y* and *X* are centered at 0 and that the POCRE algorithm sequentially constructs the orthogonal components , , …, where  and , *i* ≥ 2, is iteratively built to be orthogonal to , where *w_i_*, *i* ≥ 1, is calculated as *γ*/||*γ*||, which minimizes:(3)

subject to ||*α*|| = 1, where *g_γ_*(*γ*) is a penalty function defined by a proper regularization on *γ* with tuning parameter *λ*. Zhang et al. [5] used empirical Bayes thresholding methods, as proposed by Johnstone and Silverman [6], to introduce the proper penalty *g_γ_*(*γ*).

In this case-control genome-wide association study, the LDA is implemented with the POCRE algorithm. First, we define *Y_i_* = 1 if individual *i* is from the case population, and *Y_i_* = −1 otherwise. Second, the LDA is implemented with the threshold *c* = 0 by regressing the phenotype vector *Y* = (*y*_1_, …, *y_n_*)*^T^* against *X* using the POCRE algorithm. The design matrix *X* here contains the genotypic values of both common variants and macrovariants constructed by the PLS regression. The tuning parameter *λ* is elicited by using a testing data set with candidates from 0.8 to 0.9, with a step size of 0.01.

To prevent using the same data twice, a process that results in overfitting, we selected one out of 200 phenotype replicates and applied the PLS regression to collapse rare variants to obtain the macrovariants for each gene. We then used results from the PLS regression to analyze another phenotype replicate using the POCRE algorithm. This is not the case in real data analysis, and we suggest taking a traditional approach to splitting the data into training and testing sets, even though we may suffer a reduction in power by reducing sample size. When the sample size is a concern, we recommend using the whole data set twice, the first time for collapsing rare variants and the second time for association study, even though it may magnify the type I error.

### Data set and preprocessing

Finally, we applied the proposed methods to the binary trait in the GAW17 data. All 697 individuals were kept for our analysis after preprocessing the data using PLINK [7] for quality control. We differentiated genetic variants into three categories: SNPs with MAF ≥ 0.05, SNPs with 0.005 ≤ MAF < 0.05 but no other SNPs within the corresponding genetic regions, and macrovariants for genes with multiple rare SNPs. Three other factors—Age, Sex, and Smoke—were also used to control environmental effects. For detailed data information, see [8].

## Results

We used LDA with the POCRE algorithm to analyze each of the 200 replicates, and we report the SNPs and genes that appeared frequently across all 200 replicates. In our association study, we have two types of genetic variants: the SNPs and the macrovariants. If a SNP is found to be significant, it is reported as a significant SNP; alternately, if any macrovariants representing that gene are found to be significant, then a significant gene is reported. The frequency of nonzero estimated effects out of the 200 replicates is calculated for both SNPs and genes.

In Table 1 we list the SNPs detected in six or more replicates along with the gene in which they reside. Three of the detected SNPs lead us to the casual genes; they are C13S523 (corresponding to gene *FLT1*), C8S890 (*PTK2B*), and C6S5380 (*VNN1*). However, we noticed that only C13S523 and C6S5380 are true disease-related SNPs; C13S523 has a moderate MAF (0.066714) and a large effect size (0.64997), whereas C6S5380 has a large MAF (0.170732) but a moderate effect size (0.24437). Surprisingly, SNP C8S890, which is not in the simulation model, guides us to disease gene *PTK2B*; this finding might be due to the linkage disequilibrium between SNPs within the gene.

**Table 1:** SNPs with high frequencies of nonzero estimated effects

SNP	Gene	Chromosome	Frequency (%)
C13S523*	*FLT1*	13	41
C12S707	*PRR4*	12	6.5
C12S708	*PRR4*	12	6
C12S706	*PRR4*	12	5.5
C12S709	*TAS2R48*	12	5.5
C8S890*	*PTK2B*	8	3.5
C12S705	*TAS2R48*	12	3.5
C16S3298	*CCDC135*	16	3.5
C6S5380*	*VNN1*	6	3
C19S2864	*ZNF91*	19	3

The Gene column lists the genes in which the corresponding SNP resides. Asterisks indicate true causal SNPs.

Out of the 200 replicates, a handful of genes found through macrovariants have a high frequency of nonzero estimated effects. Both the genes and the environmental covariates detected in 10 or more replicates are listed in Table 2. Among our findings, *FLT1*, *PTK2B*, *VNN1*, and *PIK3C3* are the true casual genetic variants along with two significant environmental factors, Age and Smoke. We have noticed that *FLT1* is detected twice, once through the common SNP C13S523 and once through the macrovariants constructed by rare variants within the gene.

**Table 2:** Genes with high frequencies of nonzero estimated effects

Gene	Chromosome	Frequency (%)
Age*	NA	100
Smoke*	NA	92
FLT1*	13	17.5
PIK3C3*	18	10
*PRR4*	12	5.5
*SPHKAP*	2	5.5
*RUNX2*	6	5

Asterisks denote the true causal genes or environmental factors.

## Discussion and conclusions

Using our developed approach for GWAS, we were able to find a few true associations, but our results still suffer from limited power and a high false-positive rate. We detected the disease gene *PIK3C3* by collapsing rare variants within genes using PLS regression, and the proposed strategy may gain power through collapsing. Even though we had some success in this study, we are still concerned about the possible low power when applying this method. In fact, association studies become even more difficult when most of the causal genetic effects are due to rare variants, especially when some of them are extremely rare variants (i.e., rare alleles that are observed in only a few subjects). Compared to phenotype Q1 and Q2 in the GAW17 data, the binary trait has relatively low effect size, which makes it even more difficult to detect its signals through this research. Therefore more powerful strategies need to be further investigated in order to effectively associate rare variants with binary traits.

## Competing interests

The authors declare that they have no competing interests.

## Authors’ contributions

MZ and DZ both conceived the study and drafted the manuscript. LW performed statistical analysis and helped to draft the manuscript. VP participated in the design of the study and preprocessing of the data. YL participated in the design of the study. All authors read and approved the final manuscript.
